# Validation of preimplantation genetic tests for aneuploidy (PGT-A) with DNA from spent culture media (SCM): concordance assessment and implication

**DOI:** 10.1186/s12958-021-00714-3

**Published:** 2021-03-06

**Authors:** Baoli Yin, Huijuan Zhang, Juanke Xie, Yubao Wei, Cuilian Zhang, Li Meng

**Affiliations:** 1 Reproductive Medicine Center, Henan Provincial People’s Hospital, People’s Hospital of Zhengzhou University, Zhengzhou, 450003 Henan China; 2 Henan Joint International Research Laboratory of Reproductive Bioengineering, Henan Provincial People’s Hospital, People’s Hospital of Zhengzhou University Zhengzhou, Zhengzhou, 450003 Henan China; 3 Incinta Fertility Center, Torrance, California 90503 USA

**Keywords:** PGT, Aneuploidy, Chromosome concordance, Spent culture medium, Trophectoderm biopsy, NGS

## Abstract

**Background:**

Spent culture medium (SCM) as a source of DNA for preimplantation genetic tests aneuploidy (PGT-A) has been widely discussed.

**Methods:**

Seventy-five blastocysts that were donated for research provided a unique possibility in which multiple specimens, including trophectoderm (TE) biopsy, SCM, and paired corresponding whole blastocyst (WB) specimens from the same blastocyst source, could be utilized for the purpose of this preclinical validation.

**Results:**

To conduct a validation ploidy concordance assessment, we evaluated the full chromosomal concordance rates between SCM and WB (SCM-to-WB), and between TE and WB (TE-to-WB) as well as sensitivity, specificity and overall diagnostic accuracy. 78.67% (59/75) of NGS results in the SCM group were interpretable, a significantly lower percentage than their corresponding TE and WB groups. This discrepancy manifests itself in intrinsically low quantity and poor integrity DNA from SCM. Subsequently, remarkable differences in full concordance rates (including mosaicism, and segmental aneuploidies) are seen as follows: 32.2% (SCM-to-WB, 19/59) and 69.33% (TE-to-WB, 52/75), *(p < 0.001)*. In such cases, full concordance rates were 27.27% (15/55) in SCM-to-WB, and, 76% (57/75) in TE-to-WB *(p < 0.001)*. Collectively, the NGS data from SCM also translated into lower sensitivities, Positive Predictive Value (PPV), Negative Predictive Value (NPV), overall diagnostic accuracies, and higher Negative Likelihood Ratio (NLR).

**Conclusions:**

Our study reveals that DNA is detectable in the majority of SCM samples. Individual chromosomal aberration, such as segmental aneuploidy and mosaicism, can be quantitatively and qualitatively measured. However, TE still provides a more accurate and reliable high-throughput methodology for PGT-A. Meanwhile, cell-free DNA in SCM reporting lacks uniform diagnostic interpretations. Considering that this test is meant to determine which embryos are relegated to be discarded, PGT-A with cell-free DNA in SCM should not be permitted to be applied in routine clinical settings for diagnosis purpose.

## Introduction

A methodology increasingly used to select against embryonic aneuploidy is preimplantation genetic testing of aneuploidies with DNA from trophectoderm biopsy, recently SCM [1, 2]. These PGT-A procedures are strategies that aim to avoid chromosomally abnormal embryo transfers, thus minimizing implantation failures, miscarriages, and increasing IVF outcome. There is also ever-growing evidence that the use of TE coupled with NGS for PGT-A fails to show a positive effect on live birth rate and does not significantly improve overall pregnancy outcomes in all women, as analyzed per embryo transfer or per intention to treat [3–5]. Several issues remain unaddressed: strategies for sampling both TE biopsy and SCM, for testing and reporting, and analysis platforms that inherently overshadow current data. Additional uncertainties emanate from technical artifacts, which affect the criteria for selection of supposed viable embryos.

For TE based PGT-A, the safety of TE biopsy is still controversial. Fundamentally, TE is the external cell mass of the blastocyst that develops into the placenta and other extraembryonic membranes for implantation. However, long-term TE sequelae are presently unknown and there is insufficient ascertainment regarding TE origin and function in humans. Therefore, TE biopsy should not be undertaken as a clinical procedure, rather as an experimental procedure under approved rigorous experimental protocols in academic contexts [6]. From a micromanipulation standpoint, TE biopsy requires specific equipment and trained personnel with expertise on strict quality assessment of embryo manipulation [7, 8]. Generally, the biopsy procedure involves removing several trophectoderm cells, about 5 to 8 cells, on Day 5 or Day 6 of the embryo at the blastocyst stage [9]. The number of TE cells being biopsied and tested is relatively small due to the threat of euploid-aneuploid mosaics and chaotic abnormalities. Chaotic abnormalities with multiple aneuploids can occur in two or more cell populations that have different chromosomal makeups, which makes it difficult to ascertain the causative aneuploids. Thus, where increasing the number of biopsied cells might improve accuracy, doing so will likely reduce implantation rate so that embryologists must also factor in the need to obtain enough trophectoderm cells. Further, mathematical models demonstrate how TE biopsies cannot provide reliable information about the whole blastocyst. Quantitatively, the sample size (here, the biopsied cell number) is the key factor to PGT-A accuracy and directly relates to embryonic mosaicism and false positive and negative rates [1]. It would take at least a 27-cell biopsy, the minimal level of correct statistical representation, for one meaningful PGT-A TE. Thus, additional concerns arise about the clinical utilization of PGT-A and its accuracy; PGT-A would be invalidated without even having to consider how well TE reflects the ICM. PGS would also not be accurate enough to decide whether an embryo should be discarded [10].

Apart from the invasiveness and inaccuracy of the procedure, blastocyst mosaicism also causes TE heterogeneity, which could interfere with both accuracy and precision of the diagnoses [11]. The cell with different chromosomal components may spread randomly throughout the TE [12]. PGT-A with TE biopsy has seen additional concerns over the long-term health of its offspring; animal studies suggest that embryo biopsy could delay blastocoel formation and increase the risk of neurodegeneration and dysfunction in the offspring [13]. Likewise, since the execution of biopsy procedures have not been standardized, IVF laboratories use varied techniques in steps such as breaching the zona pellucida, separating the rest of the embryo, and determining the number of cells biopsied from the TE [9].

Given the challenges intrinsic to invasive biopsy procedures, there is increasing interest in developing noninvasive procedures. Clinically, sampling DNA from embryo spent culture media would be preferable over that from TE biopsy. Though SCM based PGT-A demonstrated some encouraging results through successfully amplified DNA and detected genomic sequences in the majority of PGT-A cases. Still, more research based on clinical outcomes is required to determine if SCM can be a reliable selection tool in PGT-A [14]. However, the SCM- based PGT-A outcomes are still subject to variation from the aspect of full concordance when compared to those obtained from whole embryos or biopsy specimens [15–17]. Nonetheless, this procedure opens a new avenue for quantitative and qualitative evaluation of chromosomal status. More importantly, SCM PGT-A would also likely increase accessibility to patients due to the nature of sequencing cell-free DNA in SCM with less invasive [14], simpler, safer, and lower PGT-A cost. We sought to evaluate whether this DNA in SCM can be liable source of information about the genetic status of the embryo.

Our current goal of preclinical validation is to ensure that SCM for PGT-A consistently achieves expected results. To this end, using blastocysts donated for research and testing extends a unique possibility in which multiple specimens, including trophectoderm biopsy, SCM, and paired corresponding whole blastocyst (WB) specimens from the same blastocyst source can serve as the positive control for comparison. Both NGS results from SCM and TE are respectively used against the same positive control so that we can measure whether the tested samples are truly representative of the WB derived from the sample. In addition, the full chromosomal concordance assessments between SCM and WB (SCM-to-WB), and between TE and WB (TE-to-WB) as well as sensitivity, specificity, and overall diagnostic accuracy were conducted. Furthermore, each individual chromosomal aberration, such as segmental aneuploidy and mosaicism, can be precisely, quantitatively, and qualitatively measured by comparing each to the positive control. This method is the most suitable and accurate validation component for measuring diagnostic reliability and encompasses all chromosomal aberrations. Thus, it is possible to sufficiently identify whether DNA in SCM will deliver a reliable PGT-A test representing its corresponding positive control.

## Material and methods

### Institutional review board approval

This study was approved by the Ethical Committee of Henan Provincial People’s Hospital. Written informed consent was obtained from all study participants. All donated blastocysts had already been diagnosed as abnormal by preimplantation genetic testing for aneuploidy by aCGH with TE biopsy. All blastocysts were donated for research under informed consent by patients undergoing IVF for treatment of infertility at Henan Provincial People’s Hospital, China. The donated embryos, related DNA samples, and data were handled anonymously.

### Sample preparation

Seventy-five blastocysts were warmed and then placed in separate 25-μL droplets of G-2 (Vitrolife, Sweden) with serum protein supplement overlain with mineral oil in Minic-1000 incubators (Cook) at 37 °C in an atmosphere of 5% O_2_, 6–7% CO_2_ balanced with N_2_. The culture drops where blastocysts (called spent culture medium) were incubated for 24-h were collected and frozen at − 20 °C for future cell-free DNA analysis. The corresponding whole blastocyst was then subjected to TE biopsy. Immediately after TE biopsy, biopsied fractions and their remaining corresponding blastocysts were washed twice, transferred into an individual lysis buffer (Yikon Genomics, China), and frozen at − 20 °C for WGA. The paired corresponding whole blastocyst served as the positive control for comparison. The culture medium, with no previous contact with the blastocyst, was incubated in the same micro droplet dish and was used as a negative control.

During protocol optimization, 20-25 μl of spent culture medium was collected from blastocyst cultured drops at 8 h (*n* = 6) at post-thaw incubation time to isolate and amplify DNA. After whole genome amplification (WGA), we observed noisy profiles following NGS, indicating low DNA yields. Therefore, the DNA sample from SCM was insufficient to produce conclusive NGS results. Similar to previous observations [16], these results do not meet necessary quality control scores for interpretation and were not included in the analysis below.

### WGA, library preparation, NGS, and data analysis

Whole-genome amplification and library preparation was performed with the use of the ChromInst (Yikon Genomics, EK100100724 NICSInst™ Library Preparation Kit) [18–20]. The sequencing was conducted with Hiseq Rapid SBS Kit v2 on an Illumina Hiseq 2500 platform (Illumina Inc., Santa Clara, CA, USA) generating 1–2 M raw reads for each sample. Initial processing of the sequence reads involved trimming the adapter sequences from the ends of the reads, which were used for sequencing library preparation, followed by filtering software to remove low-quality bases did not meet the criteria for PGS. Sequencing data were then deposited into the NCBI Sequence Read Archive under accession number PRJNA524206 (PGT-A). The high quality read numbers were aligned and mapped to the human reference genome, hg 19, and counted along the whole genome with a bin size of 1 Mb. They were then normalized by the GC content and a reference dataset. ChromGo™ Analysis Software (EK1001013, Yikon Genomics) was employed to analyze sequencing, to determine copy number variations (CNV) and to interpret NGS data. Standard PGT-A results as obtained from SCM, TE, and WB were employed. Chromosomal imbalances ≥4 Mb were defined as segmental aneuploidy in this study. When segmental or numerical aneuploidies were detected, the aberrations were always reported. In the case of mosaicism, based on the data obtained by simulating chromosomal mosaicism, we classified 23 chromosome pairs into two groups according to their chromosomal trisomy survivability. The NGS pattern detection and classification of aneuploidies was determined by copy number variation (CNV) values.

**Fig. 1:**
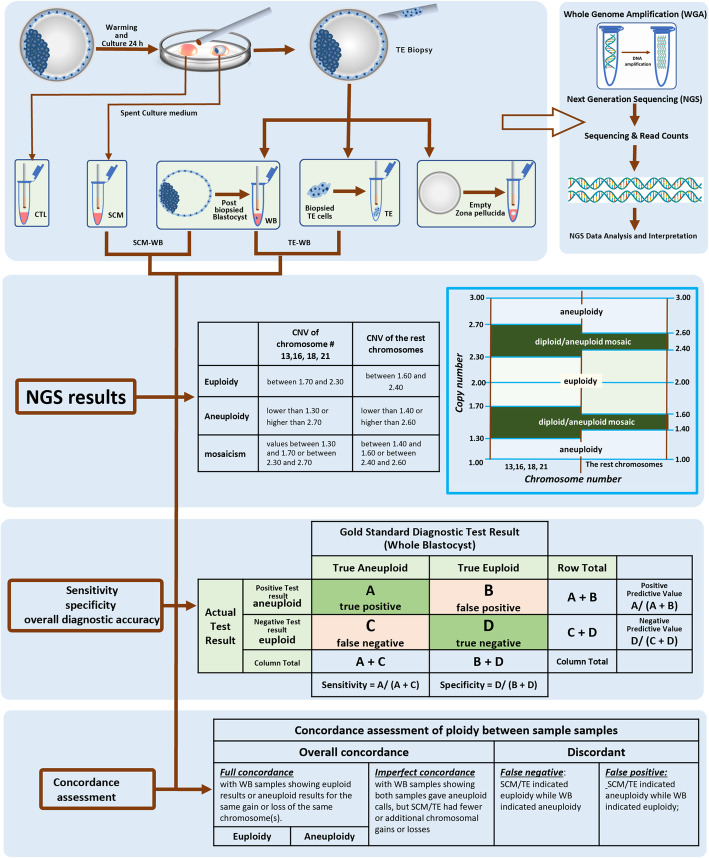
Detailed workflow for obtaining DNA samples from spent culture medium, TE biopsy and their corresponding whole blastocyst. The corresponding whole blastocysts served as the gold standard for this comparison. The samples are lysed and processed using standard clinical workflow for PGT-A. The same Whole Genome Amplified and Next Generation Sequencing from all samples were employed

For survivable trisomy aneuploidy, including trisomy error-prone chromosome numbers 13, 16, 18, and 21, CNV values between 1.70 and 2.30 were considered euploid; aneuploidy CNV values between 1.30 and 1.70 or between 2.30 and 2.70 were classified as diploid/aneuploid mosaic; CNV values lower than 1.30 or higher than 2.70 were classified as aneuploidy. This customized cut-off was established based on the reproducibility of our cell line mixtures to set the detection limit of mosaicism based on in-house validation. For the remaining chromosomes, CNV values between 1.60 and 2.40 were considered euploid; aneuploidies with CNV values between 1.40 and 1.60 or between 2.40 and 2.60 were classified as diploid/aneuploid mosaic; CNV values lower than 1.40 or higher than 2.60 were classified as aneuploidies (Fig. 1). Although the resolution of Hiseq 2500 platform NGS is validated to detect segmental (sub-chromosomal) aneuploidies of 20 Mb or larger by the manufacturer, we were able to detect segments as small as 1.0 Mb using our NGS platform and in-house ChromGo™ software. With this software, we can reliably detect the diagnostic limit down to the sub-chromosomal level. Only in the results section of the assessment of subchromosomal instability in blastocysts and reciprocal subchromosomal deletions and duplications, was the segmental CNV taken into account, but not to the exact coordinates of the variation. Similarly, mosaic chromosomes were compared in terms of their presence or absence but not in its percentage level. Due to DNA’s nature in SCM of intrinsic low quantity/abundance and poor integrity, we expect NGS data in SCM to be relatively noisier than those in TE and WB. To this end, we consider ‘aneuploidy’ to encompass both whole and segmental chromosome abnormalities for the rest of our study.

The overall diagnostic accuracy [21] of the PGT-A in the testing embryos using the summary of the probabilities are as follows: Overall diagnostic accuracy = *sensitivity* ∗ *prevalence* + *specificity* ∗ (1 – *prevalence*). Sensitivity and specificity are measures of intrinsic diagnostic accuracy based on embryonic cell-free DNA in SCM and DNA from TE biopsied cells related to the paired corresponding blastocyst, respectively. Overall diagnostic accuracy, which is the proportion of correctly classified true-aneuploidy and true-euploidy, can be reported as a global marker of accuracy [21].

### Statistical analysis

Sensitivity, specificity, negative predictive value (NPV), and positive predictive value (PPV) were used to evaluate the detection of chromosome abnormalities. The result of the whole blastocyst was considered as the gold standard. The 95% confidence intervals (CIs) of proportions were calculated by the Wilson score method. Statistical analyses were performed using MedCalc Software version 18.2.1 (MedCalc Software, 2018).

## Results

### Elimination of zona pellucida as sources of cell-free DNA in SCM

One of the major challenges for cell-free DNA assessment is potential contamination by maternal DNA from granulosa cells [22]. Regarding this challenge, we focused on optimizing procedures to ensure that cell-free DNA derives only was from corresponding blastocyst. Through DNA amplification, WGA results will give well-identified chromosomal status of the corresponding blastocyst, opening the way for clinical use of this non-invasive technology.

**Fig. 2:**
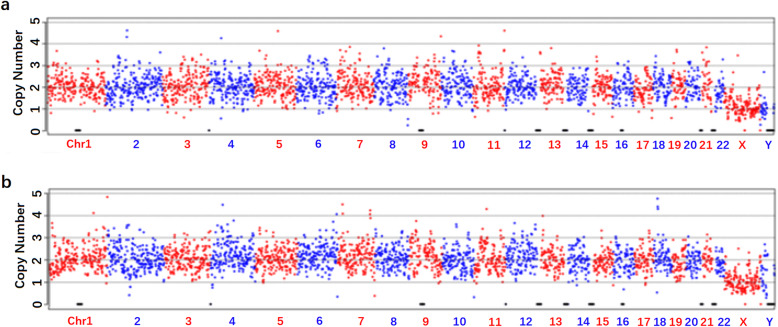
**a** Negative control I, blank medium negative controls (empty culture drop) associated with each specimen that underwent DNA amplification showed no DNA amplification in all cases. **b** Negative control II, after 24 h, cultured medium collected from the culture drop of empty zona pellucida. Each specimen that underwent DNA amplification showed no DNA amplification in all cases

To eliminate the possibility of maternal DNA contamination, we attempted to rule out the possibility of zona pellucida and associated transzonal projections as potential sources of cell-free DNA in the SCM samples. Thus, the zona pellucida (*n* = 6) was removed from the blastocyst by repeatedly pipetting with a micropipette and thoroughly washing the blastocyst. The culture media with empty zona pellucida were individually cultured for 24 h under identical culture conditions. Following NGS, negative control samples were sequenced and generated an amplification-failure pattern in blank culture media samples (Fig. 2a) and culture media with empty zona pellucida (Fig. 2b).

This observation indicates that the non-informative sample present in the control culture media with/without zona, successfully eliminated the possibility of zona pellucida and associated transzonal projections as the source of DNA in SCM.

### Overview of DNA amplification and informative NGS results in SCM, TE and WB from the same blastocyst

An overview result of ploidy is shown in Table 1. For 75 blastocysts, informative results (successful DNA amplification with interpretable NGS results) were obtained from 100% TE (trophectoderm biopsy) and WB (zona-free whole blastocysts). One of 75 (1.33%) SCM specimens showed maternal contamination. Interpretable NGS results in the SCM group were 78.67% (59/75). These results were significantly lower than their corresponding TE and WB groups. Of the NGS results per sample, there were no significant differences observed in SCM, TE, or WB aneuploid rates based on their interpretable NGS results: SCM 91.53% (54/59); TE 86.67% (65/75); and WB, 81.33% (61/75).

**Table 1:** Overview results of NGS results from SCM, TE, and the corresponding blastocyst (WB)

	SCM	TE
**Number of Analyzed sample**	75	75
**successful DNA amplification**	60 (80%)	75
**Maternal contamination**	1	0
**Interpretable NGS results**	59 (78.67%)	75
**Total Euploidy**	5 (8.47%)	10 (13.33%)
**Aneuploidy**	54 (91.53%)	65 (86.67%)

### The concordance assessment of ploidy between two groups of SCM, TE, and WB

Full chromosomal concordance assessments provide detailed measurements with more precise quantitative and qualitative comparisons of ploidy concordance assessments: first, we define the overall concordance as the sum of the total full concordance and imperfect concordance. The overall concordance rate in SCM (89.83%, 53/59) is similar to that of TE (94.76%, 71/75), (*P* = 0.335). We also define full concordance as all the same chromosomes possessing the same gain or loss between SCM and WB, or between TE and WB, where WB served as the gold standard for this comparison.

**Fig. 3:**
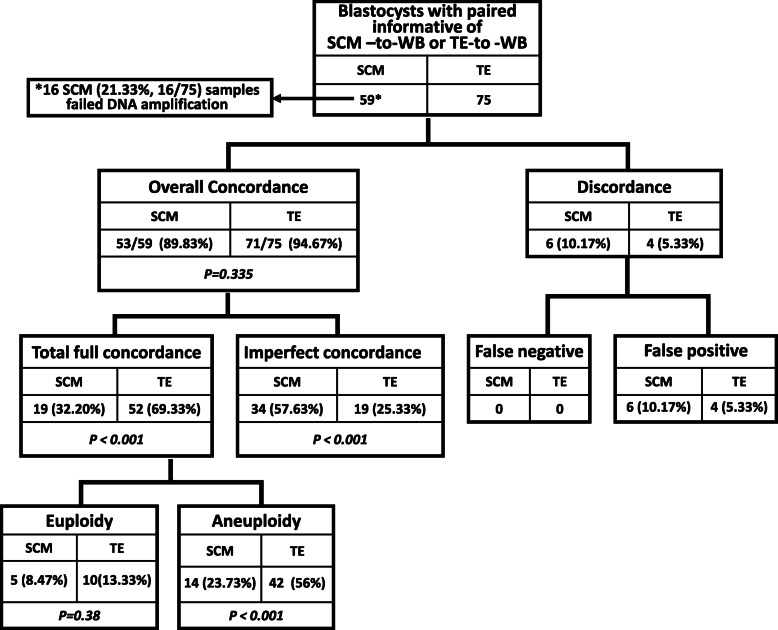
Full chromosomal concordance assessments between SCM and WB (SCM-to-WB), and between TE and WB (TE-to-WB)

Figure 3 shows the full chromosomal concordance assessments of ploidy. Remarkable differences in full concordance rates are seen, 32.2% (SCM-to-WB, 19/59) vs 69.33% (TE-to-WB, 52/75), (*p* < 0.001). Furthermore, significant inconsistent anomalies in the full concordance rate were mainly reflected in the aneuploidy, with 23.73% (14/59) in SCM-to-WB, versus 56% (42/75) in TE-to-WB (p < 0.001) Aneuploid–aneuploid imperfect concordance shows the degree of dissimilarity of aneuploidies. Imperfect concordance with WB samples indicates that comparing both SCM-to-WB and TE-to-WB gives aneuploid calls, but SCM/TE had fewer or additional chromosomal gains or losses. The imperfect concordant rate in SCM-to-WB is 57.63% (34/59), whereas it is 25.33% (19/75) in TE-to-WB, indicating that the imperfect concordant rate in the SCM-to-WB group is higher than that of TE-to-WB (*p* < 0.001). This difference furthermore demonstrates that interpretable NGS results from TE can better reflect the ploidy status of WB than what can be observed in SCM-to-WB results.

Among aneuploid–aneuploid imperfect concordant cases, a small proportion of reciprocal chromosomal/sub-chromosomal gain-loss complementary pairs were observed in the current study. These aneuploidies were detected in the two sample types among SCM, TE and WB groups, existing as complementary pairs in terms of loss versus gain chromosomally or sub-chromosomally. These complementary pairs were located either on the small (p-) or large (q-) chromosome arm, including their mosaic of their corresponding arms at the affected chromosome.

The reciprocal complementary pairs were observed in 6 blastocysts (Table 2). These pairs were located on the same or comparable chromosome arm gain and loss positions. The proportion of guanine (G) and cytosine (C) bases in the DNA molecule is usually expressed as GC content. GC base pairs are held together by three hydrogen bonds, while AT and AU base pairs are held together by two hydrogen bonds. This difference is why DNA with low GC-content is less stable than DNA with high GC-content. Among all 22 autosomes, the average of GC-content was 41.62% [23, 24]. Meanwhile, 27.27% (6/22) of autosomes had GC content below 40%. Eleven total reciprocal chromosomal gain-loss complementary pairs were identified in four chromosomes: 3, 5, 6, and 13. Coincidently, they all contained low GC content autosomes. The false positives and false negatives were classified as discordant. Discordancy is a lack of agreement between ploidy, i.e., a blastocyst is diagnosed as aneuploid in either SCM or TE, while their corresponding WB are diagnosed as euploid (false positive) or vice versa (false negative). In discordant cases, there is no remarkable difference in false positive between SCM-to-WB, 10.17% (6/59), and TE-to-WB, 5.33% (4/75), (*p* < 0.001).

**Table 2:** Reciprocal chromosomal arm gain-loss complementary pairs in SCM, TE, and WB: Aneuploid-complementary

Embryo ID	Samples	NGS results	Subchromosomal arm/mosaic	GC%
5	SEC	+ 3p(pter→p25.3,~ 11 M, ×3)	±3p	39.67
	TE	-3p(pter→p25.3,~ 11 M, × 1)		
	WB	-3p(pter→p24.3,~ 19 M,× 1)		
43	SEC	−5(×1),-13(× 1),-14(× 1,mos*),+ 15(×3,mos*),-Xq(q13.3 → qter, ~ 81 M, × 1,mos*),...	±5mos	39.51
	TE	+ 5(× 3,mos*),+ 14(p11.1 → q32.13,~ 78 M,× 3,mos*)		
	WB	+ 5(×3,mos*,~ 30%)		
46	SEC	(q24.3 → qter,~ 24 M,×1),+13q(q21.33 → qter,~ 43 M,× 3, mos*)	±13q mos	38.55
	TE	-6q(q24.2 → qter,~ 26 M,×1,mos*),-7q(q21.11 → qter,~ 81 M,× 1,mos*), −13(pter→q31.1,~ 61 M,× 1,mos*),-21 (× 1,mos*)		
	WB	-6q(q25.2 → qter,~ 17 M,× 1),+13q(q21.33 → qter,~ 44 M,× 3)		
49	SEC	+ 4(pter→q22.1,~ 90 M,× 3,mos*),-11q(q13.4 → qter,~ 64 M, × 1,mos*),-13(q21.31 → qter,~ 52 M,× 1,mos*),+ 19(× 3)	±13q- mos	38.55
	TE	+ 3(× 3,mos*),+9q(q33.1 → qter,~ 22 M,× 3,mos*),+13q(q31.1 → qter,~ 33 M,× 3,mos*),+ 15(× 3,mos*),+ 19(× 3), …		
	WB	+ 19(× 3)		
58	SEC	+ 2(p16.1 → q22.1,~ 80 M,× 3),-3(p11.1 → qter,~ 110 M,× 1),+ 6(p21.1 → qter,~ 125 M,× 3),+ 7(pter→q11.23,~ 75 M,× 3),8(p23.1 → q24.23,~ 128 M,× 1), …	±3p	39.67
	TE	+ 3(p12.3 → qter,~ 122 M,× 3)		
	WB	+ 3(p12.3 → qter,~ 122 M,× 3)		
73	SEC	+ 2(× 3),+ 4(× 3),+ 6(p22.3 → qter,~ 147 M,× 3),+ 7(× 3),+ 8(× 3),...	±6p	39.61
	TE	-6p(pter→p22.3,~ 16 M,× 1),+10p(pter→p15.1,~ 8 M, × 3)		
	WB	-6p(pter→p22.3,~ 16 M,× 1),+10p(pter→p15.1,~ 8 M,× 3)		

Comparing data of full concordances, we conclude that the TE-to-WB group provides a more accurate measurement and representation of the chromosomal constitution of the whole blastocyst.

### Sensitivity, specificity, and overall diagnostic accuracy

The metrics of sensitivity, specificity, and predictive values are often considered measures of diagnostic accuracy because they provide information on dichotomous tests, distinguishing between euploidy and aneuploidy blastocysts. From discordant aspects, both negative predictive values in TE and WB are 100%. Positive predictive values for TE and SCM are 93.85% (61/65) and 88.89% (48/54), respectively. Specificity of DNA in SCM and TE in predicting chromosomal status is based on the incidence of aneuploidy in WB. Specificity of TE (71.43%) is higher than SCM (45.45%) with sensitivity of 100% for both SCM and TE. Positive likelihood ratio in SCM (1.83) is lower than that of TE (3.50), whereas both negative likelihood ratios in SCM and TE under current study circumstances are zero. The limitation in this study is that most available donated embryos had previously been PGS tested and identified as abnormal. This fact eliminated the feasibility of investigating actual sensitivity, negative predictive value, and negative likelihood ratio. In clinical PGT-A, we expect the negative likelihood ratio to be above zero while sensitivity and negative predictive values should be less than 100%. Still, overall diagnostic accuracies in SCM-WB (89.83%) are less accurate than those in TE-WB (94.67%).

In summary, NGS data interpretation for SCM DNA is collectively translated into lower specificity, PPV, PLR, and overall diagnostic accuracies. DNA samples from TE are more stable than those in SCM. SCM is currently suboptimal for aneuploidy screening in blastocysts, but with further improvement, it remains a promising tool for non-invasive PGT-A.

## Discussion

PGT-A, though widely applied to IVF, has so-far failed to establish validated clinical utility: It reduces pregnancy chances due to the high number of false-positive diagnoses, increases additional potential negative clinical outcomes, and places financial burden on IVF patients [4]. PGT-A has been marked by an increasing number of publications, including ASRM, ESHRE, and the British Authority, as a controversial and severely questioned procedure with few clinical benefits.

SCM coupled with PGT-A (SCM-PGT-A) has emerged as a valuable option that does not have invasive risks. A plethora of validation studies in clinical IVF underline the excellent potential in SCM-PGT-A. However, it was reported [22, 25] that SCM contributes to maternal DNA contamination. Maternal DNA in SCM can cause poor concordance with corresponding blastocysts. The thin cytoplasmic projections, called transzonal projections (TZPs) [26], from cumulus cells connect to the oocyte and are crucial for normal oocyte formation. Existing TZPs within zona pellucida could either be a potential source of cell-free DNA or could interference. In our study, culture medium, which was collected from the drop where empty zona pellucida were individually cultured for 24 h, served as a testing sample to explore if zona pellucida is associated which cell-free DNA; for this reason, zona pellucida were also immediately removed following TE biopsies. The corresponding zona-free blastocysts served as the gold standard of reference in our research cohort. After test results, we eliminated the possibility of zona pellucida as a contamination source for cell-free DNA in the spent media. No further testing of zona pellucida was necessary.

It is fair to say SCM-PGT-A should only be classified as a screening method, rather than a diagnostic test. In comparison to our negative control (the empty culture drop), successful DNA amplification was observed in 100% TE and WB group samples. However, only 78.67% of attempts were interpretable. The DNA amplification QC metrics indicate the DNA sample quality from SCM is suboptimal, which is likely linked to degraded DNA in nature [1]. As diagnostic tests require both sensitivity and specificity to be as close as possible to 100%, our SCM-PGT-A results indicated acceptable sensitivity with limited specificity. Specificity rates for SCM-to-WB and TE-to-WB are 45.45 and 71.43%, respectively. These characteristics increase the risk of positive diagnosis bias, which increases the likelihood of a misdiagnoses in SCM. According to current diagnostic accuracy studies, any one of the elements in Table 3 may directly or indirectly affect the overall diagnostic accuracy through prevalence of the aneuploidy. All SCM, TE and WB samples were from the same corresponding blastocysts, minimizing the role of the aneuploidy on overall diagnostic accuracy. It is noteworthy that overall concordance characterized by dichotomy can only distinguish between euploidy and aneuploidy blastocysts. Our TE-to-WB overall concordance rate is 94.67% which is acceptable and comparable to other studies [15, 16, 27, 28]. It may reflect the genetic fact that most donated blastocysts had been PGT-A tested blastocysts with chromosome aberrations, potentially leading to high overall concordant rate. Full concordance between SCM and WB was only 32.2%, less than half of the 69% full concordance between TE and WB. This result demonstrated that SCM-PGT-A is a highly unsatisfactory test and would not be suitable to be considered for clinically genetic diagnosis. Furthermore, the imperfect concordant rate is two times higher in the SCM-to-WB group than that of TE-to-WB (57.63% vs 25.33%). Considering imperfect concordance as a source of misdiagnosis, 1/3 of embryos that currently undergo TE and 2/3 of SCM embryos will demonstrate a result that does not fully correspond to their WB pairing control. Full chromosome concordances, on the other hand, not only enhanced assessment of ploidy status, but also offered more detailed measurements with more precise quantitative and qualitative comparisons of chromosomal aberration including mosaicism, segmental aneuploidies, and a considerable number of chaotic NGS results. In our study, the full concordance rate of TE-to-WB is 69.33%. Similar findings were reported in other studies [16]. SCM overall concordance rate (89.83%) in present study is also comparable to rates that have been previously reported [16, 17] and is higher than that of Ho, et al. [15]. However, the full concordance rate of SCM-WB is 32.2%. These full concordance rates are remarkably lower than other studies [16] but are similar to this one [17]. These lower rates are likely due to the DNA sample quality in SCM. As stated in other reports, [29–31], this discrepancy is unsurprising given the likely degraded nature of the DNA in SCM.

**Table 3:** Sensitivity, specificity, and overall diagnostic accuracy

	SCM		TE
Statistic	Value	95% CI	Value
**Sensitivity**	100%	92.60 to 100.00%	100%
**Specificity**	45.45%	16.75 to 76.62%	71.43%
**Positive Predictive Value (PPV)**	88.89%	82.35 to 93.21%	93.85%
**Negative Predictive Value (NPV)**	1000%		100.00%
**Positive Likelihood Ratio (PLR)**	1.83	1.07 to 3.14	3.50
**Negative Likelihood Ratio (NLR)**	0.00		0.00
**Disease prevalence**	81.36%	69.09 to 90.31%	81.33%
**Overall diagnostic accuracy**	89.83%	79.17 to 96.18%	94.67%

Evidently, DNA quality in SCM stands out as the most limiting factor for SCM-PGT-A. NGS data analysis would have to be altered with appropriate protocol specifically for degraded DNA amplification. The interpretation of results would also have to be based on DNA intrinsically degraded low quality in SCM. Generally, commercial WGA buffers are designed for TE samples in small volumes. Yet, the SCM sample from the culture drop has a relatively larger volume, which requires reaction components to be scaled up. This requirement not only increases costs, but also necessitates additional validation and optimization. A reduced volume (12 μl) of blastocyst culture media [20] could certainly concentrate DNA in SCM, but deviates from the manufacturer’s recommendations. Reducing the volume would therefore require further validation so that the blastocyst’s developmental potential is not compromised. In our study, only a small proportion of reciprocal chromosomal/sub-chromosomal gain-loss complementary pairs were observed in imperfect concordant cases. Alternative criteria to classify embryos as mosaic would, biologically, require the presence of reciprocal aneuploidy in two different cells/cell lines or two different biopsied samples from the same embryo [32]. The frequency of whole chromosome or sub-chromosomal arm gains and losses should be similar. For example, take one biopsy that displayed a monosomy (2n-1) of a specific chromosome and another biopsy from the same embryo displaying trisomy (2n + 1) for the same chromosome [29, 33]. It is expected that at least one embryo should display reciprocal errors for at least one chromosome. Indeed, in our study, we observed 11 reciprocal chromosome arm loss-gain complementary pairs located in four different chromosomes in six blastocysts, which is similar to other observations [12, 22]. Such findings exhibit shared complementary aneuploidies. Coincidentally, in the current study, all four chromosomes associated with reciprocal gain-loss complementary pairs were low GC-content chromosomes. Studies on human-inherited diseases and cancers also revealed that DNA breakpoints tend to occur in DNA sequences with low GC content [24]. Due to small sample size, we cannot robustly ascertain whether a low GC-content sequence would be more vulnerable to DNA breakpoints than a high GC-content sequence. However, utilizing GC content and reciprocal sub-chromosomal arm gain-loss complementary as a reference may prove a more efficient tool in distinguishing real DNA segments from NGS data noise generated from a sample of DNA in SCM [23, 34].

**Fig. 4:**
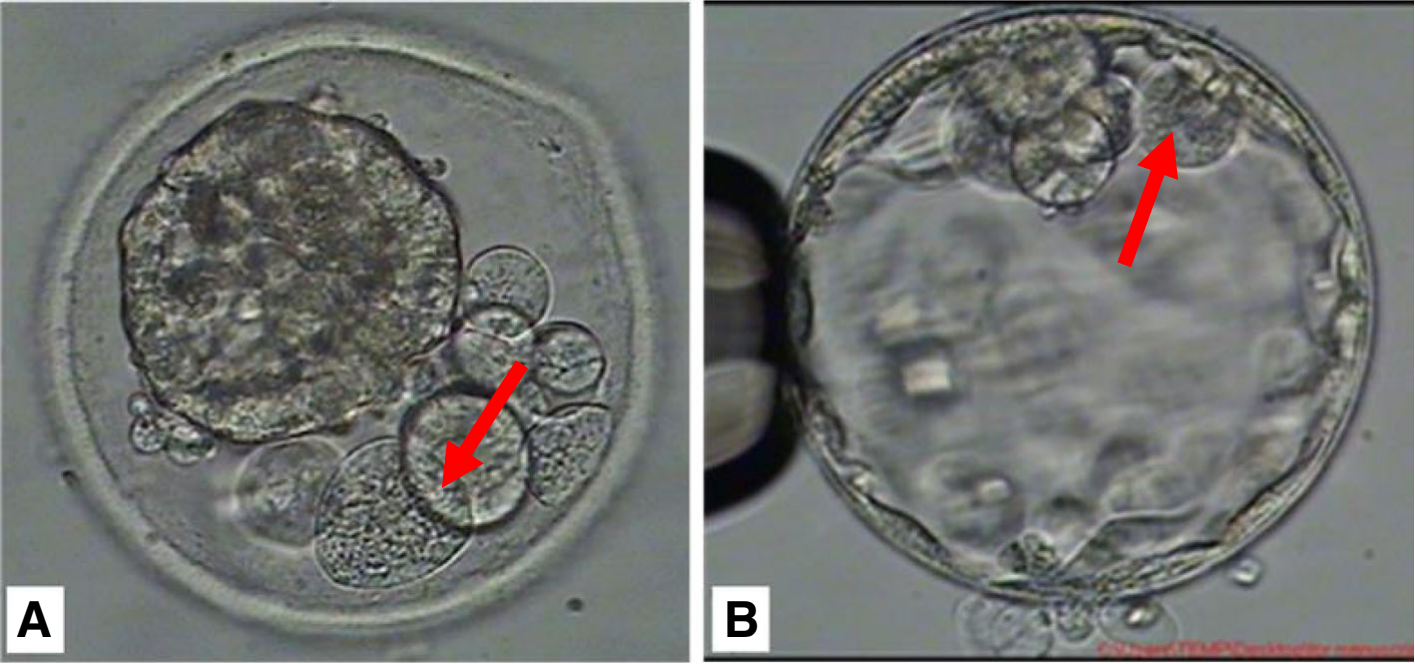
**a** Embryonic cells phagocytosed by neighboring cells or expelled into the perivitelline space or **b** which into the blastocoel cavity

To date, several studies have provided critical insights into the fact that blastocyst mosaicism can only be reported based on a single TE biopsy and has been ascribed to 2–13% of embryos tested using NGS. Contrarily, data from NGS studies disaggregating whole embryos suggests that mosaicism may be present in up to ∼50% of blastocysts [35]. An embryo that displays mosaicism, reciprocal aneuploidy, and self-corrective mechanisms is unlikely to yield true data. We are also unable to confirm if DNA in SCM is released due to cell apoptosis or necrosis death in the blastocyst. In our daily clinical laboratory, apoptotic cells may be either phagocytosed by neighboring cells, or expelled into the perivitelline space (Fig. 4a) or blastocoel cavity (Fig. 4b) [35, 36]. This observation is similar to what Orvieto et al. reported, where they observed the ability of human embryos to potentially self-correct by eliminating abnormal blastomeres as cell debris/fragments [37]. Based on published survey data [4, 38]., there have likely been at least 400 live births that were obtained by transferring embryos designated by PGT-A as “abnormal-aneuploid” or “mosaic” Considering physiological consequences of aneuploidy embryonic cells in the blastocyst, self-corrective mechanisms are likely based on chromosome stoichiometry [39]. Cell functional physiology also depends on a balanced dosage of gene products. Due to the alteration of chromosome dosage in aneuploidy cells, RNA and protein-level expression of genes carried by the aneuploid chromosome influences the transcriptome and proteome of the cell. Consequently, the effects of aneuploidy on the transcriptome- and proteome-generated cellular stress states such as lagging chromosomes, homologous chromosome nondisjunction, replication stress via stopped or delayed replication fork progression. In terms of metabolic stress, an unbalanced dosage of genes in aneuploidy embryonic cells can alter ROS levels in the mitochondria, which can induce DNA damage and apoptosis. This imbalance would lead to negative feedback, perpetuating aneuploidization by until the aneuploidy is eliminated as a self-corrective consequence. Such a reaction leads aneuploid cells in the blastocyst to decline in number as development progresses, ultimately resulting in a normal fetus. This could explain why “abnormal-aneuploid” or “mosaic” blastocysts have the capacity to develop normally and have sparked discussions regarding the ability of embryos to self-correct. These discussions raised the question of how the best diagnostic techniques and technologies could be successful in determining down-stream self-correction.

This chromosomal mosaicism can also be observed in several normal human tissues [39] suggesting that it is a normal, rather than pathological, feature of stem cell lines [40]. Unfortunately, chromosomal mosaicism would be a perpetual diagnostic and clinical dilemma in PGT-A. Therefore, while systematic implementation in clinical practice may appear unjustified, chromosomal mosaicism should be considered a research tool, and should only be offered with caution and appropriate informed consent.

## Conclusion

In this study, the full chromosome concordances of SCM, TE and whole blastocyst contribute experimental evidence to the validation of PGT-A at the blastocyst stage. Due to intrinsically low quantity/abundance and poor integrity associated with DNA samples in SCM, it resulted in diagnosis biases. Subsequently, the results negatively contributed to our full concordance assessment so that the full concordance rate between TE and WB was greater than that of SCM and WB. Because of this bias, we conclude that SCM provides less accurate and reliable high-throughput methodology for PGT-A. Although cell-free DNA in SCM has the potential to represent a safe and simple strategy in PGT-A, it still requires further validation. This procedure works as expected, consistently achieves expected results, but is not comparable to currently used tests for TE biopsies. Therefore, it is a rule-in test, as opposed to a rule-out test [41] and could be used for optimizing noninvasive embryo prioritization. With further improvement, it remains a potential tool for noninvasive PGT-A. The diagnostic efficiency of cell-free DNA in SCM may ultimately require custom-tailored design methodology for sampling strategies, tailored blastocyst culture media components, sample storage to prevent the length of the DNA in SCM sample from further degradation, and new WGA techniques for the efficient amplification of degraded/short DNA fragment samples.

## Data Availability

The datasets used and/or analyzed during the current study are available from the corresponding author on reasonable request.

## Abbreviations

PGT-A: preimplantation genetic tests for aneuploidy
SCM: spent culture media
TE: trophectoderm
WB: whole blastocyst
WGA: whole genome amplification
CNV: copy number variation
Cis: confidence intervals
NGS: next generation sequencing
PPV: Positive Predictive Value
NPV: Negative Predictive Value
PLR: Positive Likelihood Ratio
NLR: Negative Likelihood Ratio
TZPs: transzonal projections
G: guanine
C: cytosine
